# What is important, what needs treating? How GPs perceive older patients’ multiple health problems: a mixed method research study

**DOI:** 10.1186/1756-0500-5-443

**Published:** 2012-08-16

**Authors:** Ulrike Junius-Walker, Jennifer Wrede, Tanja Schleef, Heike Diederichs-Egidi, Birgitt Wiese, Eva Hummers-Pradier, Marie-Luise Dierks

**Affiliations:** 1 Institute of General Practice, Hannover Medical School, Hannover, Germany; 2 Institute of General Practice, University Medical Center Hamburg-Eppendorf, Hamburg-Eppendorf, Germany; 3 Institute of Biometrics, Hannover Medical School, Hannover, Germany; 4 Institute of Epidemiology, Social Medicine and Health System Research, Hannover Medical School, Hannover, Germany

**Keywords:** Health priorities, Multimorbidity, Old age, Family practice, Patient-centred care

## Abstract

**Background:**

GPs increasingly deal with multiple health problems of their older patients. They have to apply a hierarchical management approach that considers priorities to balance competing needs for treatment. Yet, the practice of setting individual priorities in older patients is largely unexplored. This paper analyses the GPs’ perceptions on important and unimportant health problems and how these affect their treatment.

**Methods:**

GPs appraised the importance of health problems for a purposive sample of their older patients in semi-structured interviews. Prior to the interviews, the GPs had received a list of their patients’ health problems resulting from a geriatric assessment and were asked to rate the importance of each identified problem. In the interviews the GPs subsequently explained why they considered certain health problems important or not and how this affected treatment. Data was analysed using qualitative content analysis and quantitative methods.

**Results:**

The problems GPs perceive as important are those that are medical and require active treatment or monitoring, or that induce empathy or awareness but cannot be assisted further. Unimportant problems are those that are well managed problems and need no further attention as well as age-related conditions or functional disabilities that provoke fatalism, or those considered outside the GPs’ responsibility. Statements of professional actions are closely linked to explanations of important problems and relate to physical problems rather than functional and social patient issues.

**Conclusions:**

GPs tend to prioritise treatable clinical conditions. Treatment approaches are, however, vague or missing for complex chronic illnesses and disabilities. Here, patient empowerment strategies are of value and need to be developed and implemented. The professional concepts of ageing and disability should not impede but rather foster treatment and care. To this end, GPs need to be able to delegate care to a functioning primary care team.

**Trial Registration:**

**German Trial Register (DRKS):** 00000792

## Background

General practitioners (GPs) attend to the multiple health problems of older patients. Patients from 70 years onwards have on average 8–12 conditions affecting their health and well-being, everyday life activities and social participation [1]. Many conditions are chronic and involve continuing care. Dealing with these simultaneous problems is a daily challenge for GPs [2]. They feel overwhelmed [3] and find it difficult to balance competing needs and to decide upon treatment priorities [4]. Guidelines do not facilitate this prioritisation process as they refer to treatment options of single diseases and potentially harm patients if simply added up [5]. In daily practice, GPs manage multiple health problems by falling back on an intuitive process of priority setting for treatment [6].

Priority setting is usually based on the subjective value of importance [7,8]. In the general practice setting, the importance that GPs and their patients attach to health problems will therefore have an impact on the decision of whether to prioritise them for treatment.

The need to set treatment priorities in patients with multiple diseases is generally recognised [9]. Yet, how this complex decision-making process is conducted and which values, incentives and obstacles influence it, has hardly been investigated. There is some indication that doctors tend to underestimate the importance of health-related everyday life issues for older patients [10-12] mainly due to the influence of the biomedical paradigm [13,14]. We want to explore whether this position and other reasons define the perception of importance and affect the determination of treatment priorities.

This paper will focus on the GPs’, and not the patients’, views. As GPs tend to be paternalistic in the decision-making process for treatments of older patients [15], our primary interest lies with the disclosure of their reasons. In a mixed methods approach we investigate

(1) the reasons GPs give when appraising the importance of their patients’ health problems,

(2) whether the GPs’ reasoning in this process is dependent on the nature of the underlying health problem or not,

(3) how the perceived importance and the nature of the underlying health problem relate to active treatment statements.

We interviewed GPs in Germany, who generally work single-handedly in a competitive situation as self-employed doctors. They only work in loose co-operation with community-based specialists and other health care providers. Compared with other European countries, German GP consultations are distinguished by high patient contact rates and short consultations [16].

## Methods

GPs were given a comprehensive list of health problems for each participating older patient generated from a geriatric assessment. They subsequently rated the importance of each problem. These ratings formed the basis of the semi-structured interviews, in which the GPs gave their reasons for their ratings.

### Recruitment

JW and HDE conducted the interviews between September 2008 and January 2009. Nine GPs and 35 patients took part. To gain a purposive sample of GPs stratified by sex and location (rural/urban), we recruited five respondents from a sample of 30 GPs who were contacted by written invitation and chose four more GPs through professional contacts.

The practice nurses consecutively enrolled patients entering each practice, irrespective of their reason for contact, after 10 a.m. on predefined weekdays. We intended to interview four patients per practice, one male and one female patient from each of the two age groups 70–80 years and over 80 years. In total 35 out of 48 contacted patients (73%) agreed to participate.

Participating doctors and patients were informed about the study and gave written consent. The Ethics Committee of the Hannover Medical School approved the study (No 5096, 2008).

### Data collection

A study nurse administered the computer-aided geriatric STEP assessment to every patient in each practice to gain an overview of their health problems. STEP was developed in a European Concerted Action to obtain a comprehensive view of older patients’ health issues. It consists of 38 question items and eight examinations/laboratory findings (blood pressure, arrhythmia, fasting glucose, cholesterol, TSH, foot examination, timed up and go test, clock-drawing test). Items are allocated to 10 health domains: physical conditions, pain, senses, functional disability, social participation and finances, medication use, cognitive function, mood, lifestyle, and immunisation [17].

Immediately after the assessment, the study nurse went over the computer-generated list of disclosed problems with the patient. The GP also received this list. Patient and GP then independently rated each problem according to its importance on an ordinal scale (‘not’, ‘slightly’, ‘rather’, ‘very important’).

A few days later, JW and HDE interviewed the GPs using the problem list with the doctor’s importance ratings and the initially blinded patient ratings. For each problem the doctors were asked: “Why is this problem important/unimportant to you?” After their explanations, the patients’ ratings were disclosed. The doctors were then invited to talk about their immediate thoughts.

### Qualitative analysis

**Figure 1:**
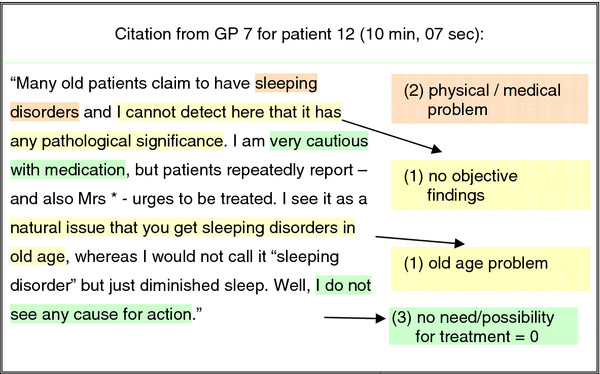
**Coding of citations – an example.** (1) reasons for unimportance: inductively developed categories. (2) dedicated health domain. (3) declaration of treatment on a scale: (−1) no declaration, (0) no treatment, (1) treatment.

All 35 interviews were audio-recorded and transcribed verbatim. JW and HDE independently marked all quotations on the importance of health problems. Qualitative content-analysis involved paraphrasing, reducing and abstracting the GPs’ quotations to inductively develop categories in an iterative approach. TS and UJW revisited all quotations to assign to each one: (1) one or more categories according to the reason for its importance (with a primary reason marked if several reasons were given), (2) the appropriate health domain, and (3) any statement about treatment scaled according to ‘no statement’, ‘no treatment necessary or possible’, ‘treatment intended or continued’ (Figure 1). Any disparate opinions were discussed in the team with MLD.

### Quantitative analysis

All quotations were fed into an SPSS data sheet, version 18. Each quotation formed a data case and was related to the interviewed doctor, to its patient, to its health domain, to the doctor’s dichotomised importance rating (important = rather or very important; unimportant = not or slightly important) and to its treatment statement.

Since 30% of quotations had two or three explanatory categories of importance, we were interested in the overlap and calculated two tetrachoric factor analyses, one with the 14 categories of importance and one with the 16 categories of unimportance. The factor analyses reduced the number of categories and contributed to the meaningful aggregation of categories into themes. We did not expect the two models to fulfil the standards of internal consistency: Firstly, GPs only gave a maximum of 3 simultaneous statements for the declaration of a problem as important or unimportant (in many cases they gave only one), which may have limited the correlation between different statements and, therefore, the explained variance. Secondly, our only intention was to pool categories into generic terms without any causal explanation.

To examine whether the specific themes for importance and unimportance were dependent on the nature of the underlying health problem, they were cross tabulated.

In a multilevel logistic regression model, we explored the extent to which the nature of the problem and the doctor-perceived importance predict active treatment statements (initiate, continue, change or monitor). For this purpose, all quotations with their health domains and their status of perceived importance were entered into the model as well as age and sex of GPs and patients (fixed effects). Because of the nested data structure, patients and doctors were entered as random effects.

## Results

We recruited GPs with a variety of personal and practice characteristics. Rural practices had more patient contacts compared to urban practices but relatively fewer patients of 70 years and older. The patient sample was balanced for gender and age. The older age group (80 years+) had a median of 16.5 problems as compared to the younger age group (median 14). Female patients disclosed more problems than male (Table 1).

**Table 1:** GP, practice and patient characteristics

**Patient characteristics**	**Female**	**(IQR)**	**Male**	**(IQR)**
Number	18		17	
Age group (70–80 / 80+)	10 / 8		9 / 8	
Education index (low / medium / high)	6 / 12 / 0		1 / 10 / 6	
Median number of health problems	19	(13.0-29.5)	14	(10.0-16.5)
Worries about health (N)	10		6	
**GP characteristics**	**Female**	**(range)**	**Male**	**(range)**
Number	4		5	
Median age (years)	47.5	(43–60)	48.0	(46–55)
Specialty training: GP / internal medicine	3/1		3/2	
Median practice experience (years)	15.5	(11–22)	11	(7–14)
**Practice characteristics**	**City**	**Small town**	**Country**	**(overall range)**
Number of practices (N)	3	3	3	
Single handed practices (N)	1	2	2	
Median size (patient contacts quarterly)	950	1000	1200	(700–1800)
Median% of patients >70 years	36	30	25	(10–45)

IQR = Interquartile range.

158 explanations for the importance and 147 for the unimportance from a total of 634 patient problems could be evaluated. We did not obtain reasons for all uncovered patient problems, because GPs either combined some related problems in their explanations, or they were uncertain about some reasons, or in some cases skipped explaining problems. In the case of 34 patient problems, GPs gave explanations both for the importance and unimportance. This was the case when they elaborated on discrepant doctor-patient views.

We will first present the themes of the reasons given for rating problems as important or unimportant (resulting from the two factor analyses). As explicated above, we did not postulate a high internal consistency for our models, and indeed the KR-20 coefficients with maximum values of 0.35 were low, whereas factor loadings exceeded a level of 0.4, which is considered as sufficient for an interpretation. Subsequently, we will show that GPs’ reasons are dependent on the nature of underlying health problems. And lastly, we will demonstrate that both the GPs’ importance ratings and the nature of health problems predict active treatment statements.

### Why GPs find some health problems important

**Figure 2:**
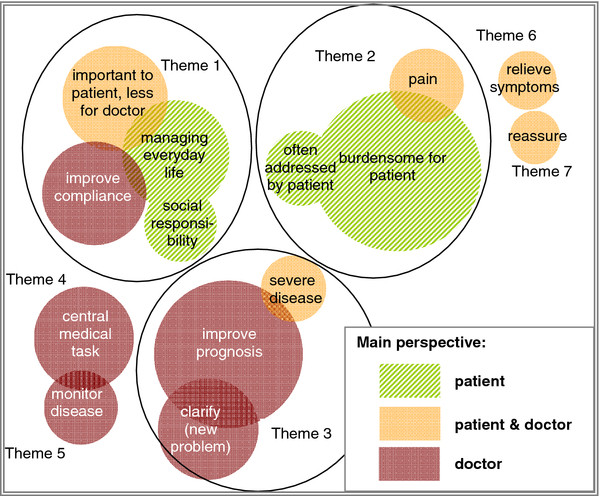
**Themes for important problems.** The circles represent the categories inductively developed by qualitative content analysis. The size of the circles is proportional to the frequency of quotes in this category. The colour indicates the perspective that GPs tend to assume in this category. Themes identified: Theme 1: GP adopts patient’s view on the importance of a problem, but cannot assist. Theme 2: doctor is empathetic. Theme 3: doctor is active. Theme 4: central medical task. Theme 5: monitor disease. Theme 6: relieve symptoms. Theme 7: reassure.

The interviewed GPs used seven themes when explaining their reasons for their importance ratings. Whereas three themes derived from the aggregation of categories into factors, four categories remained and could not be related in this way. Figure 2 illustrates the identified themes detailed below and indicates which position doctors adopt (the patient’s, the doctor’s or both), when explaining the importance of health problems.

### Theme 1: GP adopts patient’s view on the importance of a problem, but cannot assist further

The GPs commented that some cardiovascular, hearing and vision problems were more important to their patients than to themselves. In their view, patients were a little too absorbed with these conditions, as they were not really serious or were already well managed. Other problems related mainly to difficulties patients experienced in their everyday life or as a carer. Again the doctors were well aware of the patients’ assessments of their situation, but considered them either not vitally important or inexorable in their progression.

"D4.P1 *…and that I know that it is important [for the patient] that he cannot walk properly… We can try to get him up the three flights of stairs, but in principle one cannot change anything.*"

Doctors referred to a more autonomous patient type when relating to the important need for patient-compliance. Some complained that the patients did not “accept” and “implement” or “trivialised” doctors’ recommendations. Yet the GPs often understood the patients’ differing motives, specifically anxiety, different priorities or cognitive limitations.

### Theme 2: GP is empathetic

Some health problems were important to doctors, because they sympathised with the patients’ situation. Due to repeated patient visits GPs were often well acquainted with the problem. They conveyed their empathy with phrases, such as “dreadful”, “burdensome”, “threatening”, “hefty” and “exasperating”. GPs were also sympathetic to patients’ worries and anxieties. However, treatment options were seldom mentioned.

"D3.P35 *This [macula degeneration] makes her very sad…. There is not much that can be done about it.. She made inquiries everywhere;… and, well, someday she will be dependent on an assistance.*"

### Theme 3: GP is active

Some patient problems had a severe prognosis with loss of autonomy or even death and which required attentive and careful treatment. Other problems involved some uncertainty, and it was important to pursue this. Finally, cardiovascular problems gained the GPs’ full attention. They talked about “risk management” and “risk profiles”, “complications” and “vital threats”.

"D4.P1 …*Because he has had a heart attack, and I tell myself…be watchful, it is important to pay attention. But I think for him [the patient] this [episode] is over.*"

**Themes 4–7: Reassuring patients** that conditions are harmless and **relieving symptoms** when patients suffer from pain were hardly mentioned as primary, but sometimes as additional, reasons for importance. Doctors regretted that these conditions are not sufficiently treatable.

**Monitoring diseases** was often used as a secondary reason for importance. For example, controlling blood pressure or lab parameters was perceived as an essential routine. Problems relating to a **central medical task** were often cardiovascular, respiratory conditions and diabetes. Although well controlled, the doctors regarded them as vital.

"D3.P34 For us both her blood glucose is [important]… Mrs * is very conscientious and has her glucose under control;… and [she] keeps our agreements."

### Why GPs find some health problems unimportant

**Figure 3:**
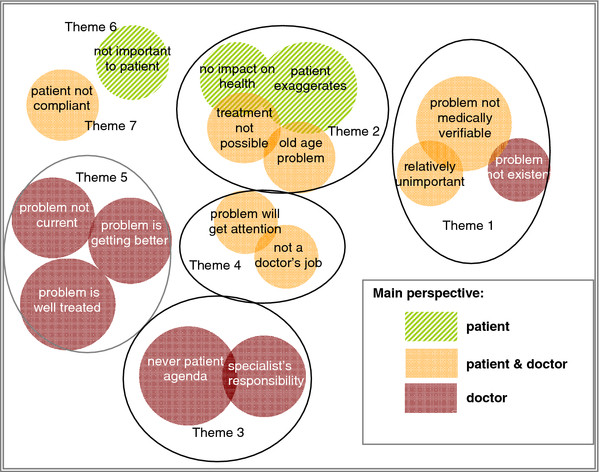
**Themes for unimportant problems.** The circles represent the categories inductively developed by qualitative content analysis. The size of the circles is proportional to the frequency of quotes in this category. The colour indicates the perspective that GPs tend to assume in this category. Themes identified: Theme 1: no need for further attention. Theme 2: doctor is fatalistic. Theme 3: doctor has no mandate. Theme 4: not a doctor’s responsibility. Theme 5: problem well under control. Theme 6: not important to patient. Theme 7: patient is not compliant.

Again seven themes were determined for problems that doctors found unimportant. Four themes originated through factor analysis. One more theme was ascertained by merging three categories because of their similar content. These categories usually provided quotations with only one explanation and therefore could not be associated using factor analysis (Figure 3).

### Theme 1: No need for further attention

The GPs used negative objective findings (e.g. lab results, cardiac tests) to reinforce the unimportance of problems. Doctors felt a bit pressured by some patients who would not fully accept these findings and expected further clarification. Other problems were newly uncovered by the assessment, and doctors questioned their existence. Finally GPs explained that some problems did not need attention because they were unimportant compared to more severe ones.

"D5P10 Problem with doing housework: *“Because of his pre-existing conditions he is severely impaired, so that I do not find it important that he can achieve a lot at home…He should go for a walk rather than work in the garden.”*"

### Theme 2: Doctor is fatalistic

Doctors felt fatalistic because some problems seemed to have little impact on patients’ lives but were often broached. Age-related problems affected their patients more seriously but were classified as unavoidable: “it is normal for this age”, the patient is “entitled to it”. Doctors also revealed a certain helplessness on how to act: “one has to accept this”, and “one has to see how to deal with this, what can be done”.

"D10P43: *“The patient often reports dizziness. But this is for her every-day life and consequently for my treatment subordinate because there is nothing that you can tackle, neither the cardiovascular system nor the [local] perfusion or other causes. This is something which relates to ageing.”*"

### Theme 3: No mandate

For some newly uncovered problems, e.g. taboo subjects or sleeping disorders, GPs assumed that patients coped; otherwise they would have already consulted them with this agenda. Impaired senses or chewing difficulties were directed to specialists’ care.

### Theme 4: Not a doctor’s responsibility

GPs were often not aware of housing or financial problems, or loneliness. On hearing this, they felt that they could not take matters into their own hands, and directed the responsibility to a family member.

### Theme 5: Problem well under control

The GPs mainly referred to cardiovascular risk factors or conditions when they reported that treatment parameters were good and patients compliant. Functional problems were cared for adequately. Some problems were getting better or disappeared altogether.

**Theme 6** dealt with “**GP adopts the patient’s view on the unimportance of a problem**”. Doctors felt, however, that patients denied there was a problem or were not aware of its relevance. The GPs showed even more resignation when talking about the **“non-compliant patient”** who made no treatment progress (**theme 7**).

"D8.P31 *I know with his sugar he is very erratic; insofar I think this is not important to him (laughs). Well, yes, [I have] spoken about it, why is this so elevated…He is just unconcerned, does not care about it.*"

### How GPs’ reasoning depends on the nature of the underlying health problem

All quotations were categorised according to the reasons given for the problems’ ratings and their underlying health domains (Tables 2 and 3). Important problems that affected everyday life (pain, senses, functional disability, housing/finances, mood) predominantly generated awareness and empathy. Everyday life problems and lifestyle issues, which were rated as unimportant, had induced fatalism or delineation of responsibilities.

**Table 2:** GPs’ primary reasons for importance stratified according to the nature of patients’ problems

Doctor (is)…reasons (N)*	**Adopts patient’s view on importance of problem, but cannot assist**	**Emphatic**	**Active**	**Re-assures**	**Considers as central task**	**Monitors disease**
**Physical**	14	13	26	0	12	2
**Pain**	2	9	3	0	0	0
**Senses**	3	2	2	0	0	0
**Functional disability**	12	9	0	0	0	0
**Housing & finances**	6	1	0	0	0	0
**Medication**	2	1	4	0	1	0
**Cognition**	1	0	3	0	0	0
**Mood**	4	7	4	1	1	0
**Lifestyle**	2	0	2	0	1	0
**Immunisation**	2	0	3	0	3	0

*158 primary reasons are grouped into 6 themes the 7^th^ theme “alleviate symptoms“ did not occur as a primary reason.

**Table 3:** GPs’ primary reasons for unimportance stratified according to the nature of patients’ problems

Doctor sees (is)…reasons (N)*	**No need for attention**	**Fatalistic**	**No mandate**	**No responsibility**	**Adopts patient’s view**	**Patient not compliant**	**Well treated problem**
**Physical**	15	11	10	1	7	3	23
**Pain**	1	3	0	0	0	1	2
**Senses**	5	2	5	0	1	0	0
**Functional disability**	3	7	2	1	0	0	0
**Housing & finances**	0	3	0	5	0	0	1
**Medication**	3	3	0	0	0	0	2
**Cognition**	1	0	0	1	1	0	0
**Mood**	0	3	1	3	0	0	1
**Lifestyle**	1	4	0	0	0	1	1
**Immunisation**	0	2	1	0	5	1	0

*147 primary reasons are grouped into 7 themes.

Medical problems (medication, cognitive function, physical conditions), although well controlled, were thought to be important because monitoring and treatment was required. They were also considered unimportant because they were well controlled. Thus it seems that the perceptions of importance or unimportance were sometimes two sides of a coin influenced by the doctor’s locus of control (Table 2 and 3).

### How the perceived importance of health problems affects treatment

A prominent finding pertains to the close link between importance and professional action within the GPs’ quotes. 70% (199/273) of the reasons given for importance also dealt with considerations about what is, and should be, done, or what cannot be done. In 63% (125/199) of these accounts, treatment was deemed unnecessary, not possible, vague, or was to be reduced. The rest represented active treatment through monitoring, continuing, initiating or changing interventions.

This close link is investigated further using a multilevel regression model because it is the doctors’ treatment suggestions which directly affect patients. Additional file 1 displays the preparatory bivariate findings of the variables for the model. Table 4 presents the predictors for active treatment statements. The physical nature of a health problem (OR 9.8) or a medication issue (OR 9.9) predicted active treatment most strongly, followed by the doctor-perceived importance of the problem (OR 3.4).

**Table 4:** Multilevel logistic regression model with active treatment as the dependent variable

**Predictors for active treatment**	**Odds ratio**	***p***	**95% CI**
Problem considered important by doctor	3.44	<0.01	1.54 -7.71
Health domain: vaccination (reference)	1.00		
Physical problems	9.75	<0.01	1.79 -53.17
Pain	0.91	0.93	0.09 -8.64
Senses*	-		
Functional disability	0.27	0.33	0.02-3.82
Housing & finances	0.38	0.48	0.03-5.51
Medication	9.94	0.03	1.23 -80.27
Cognition	2.41	0.50	0.19 -30.68
Mood	1.58	0.65	0.22-11.59
Lifestyle	3.38	0.33	0.29-38.82
Doctor’s age	0.94	0.18	0.85-1.03
Doctor’s gender	0.96	0.95	0.34-2.71
Patient’s age	0.98	0.62	0.91-1.06
Patient’s gender	0.73	0.43	0.33-1.61

* No active treatment statement for problems in the health domain ‘senses’.

## Discussion

GPs were interviewed about their underlying reasons for their appraisal of the importance of health problems in older patients with multimorbidity. A qualitative analysis reveals that GPs give several reasons for the importance of health problems. Somewhat simplified, two frequent patterns emerge: first, the relevance of treatable and severe physical disease, and, second, adopting the patient’s perspective and showing empathy. For unimportance two contrasting reasons were apparent: no further attention required for well-controlled or less severe physical problems, and no further action possible especially for geriatric syndromes, psychosocial and functional issues. The perceived importance of a problem is closely related to its treatability. Here, physical problems fare better than functional and social issues.

### How GPs explain the importance of patient problems

The rationale behind “importance” is dependent on the following conditions: adopting the patient perspective and being empathetic, the clinical relevance of the health issue, and the doctor’s own personality attributes.

Adopting the patients’ perspectives and empathy are prerequisites for patient-centred treatment and patient involvement. They facilitate a cognitive and emotional connection and establish common grounds for such actions [18,19]. Whereas our doctors show understanding of the patients’ problems, they find it difficult to act upon this understanding - possibly because the scope of actions, such as patient involvement and empowerment is not sufficiently recognised.

Health issues with clinical relevance are also deemed important. In this case, the GPs’ reasoning is based on what is medically possible. Medical expertise is an essential part of medical professionalism and moreover prized by the patient [20]. However, the danger lies in GPs equating medical professionalism with medical expertise, so that patient issues may be condensed to purely what is clinically relevant.

The different personality traits of doctors may explain why the importance of similar patient issues is rated with variance. The GP’s perception of control may influence whether a medical problem is seen as unimportant despite or even due to being well controlled or whether a functional issue is seen with empathy or fatalism. There are indications that character traits entail such variations in medical care [21,22].

### How GPs relate explanations on importance to professional action

GPs linked the majority of explanations on (un)importance to treatment considerations. This is also how GPs handle patient problems in the consultation. Consultations are concluded with doctors’ advice and treatment.

More so than the perceived importance of a patient problem, physical and medication issues predict active treatment. Functional disabilities, problems with the senses and social circumstances, however, tend to be associated with no action. Explanations for this phenomenon are found in our qualitative analysis:

GPs talk about physical issues, such as verifiable diseases, cardiovascular risk factors and medication issues with confidence, and carry out committed expert treatment. This expertise is facilitated by tools, such as evidence-based guidelines and disease management programmes; it is accredited within the profession and paid within the health system [23].

A different picture emerges when GPs talk about geriatric problems, e.g. sleeplessness, dizziness, incontinence, pain as well as functional problems with everyday life and with hearing and vision. They explicate the inexorable progression of these chronic problems. The impact on patient management is seen as restricted: there is no cure, relief is partial and sometimes outside the scope of responsibility.

### Our GPs have dealt with these chronic geriatric and functional problems in three ways

First, they expect the patients to cope with the recurrent, advancing or disabling nature of such problems themselves. Psychological adjustments to chronic disease are necessary for successful ageing [24]. Indeed there is evidence that expressing one’s emotions helps adapting, and is used in psychological interventions. Cognitive-behavioural and self-management strategies have also shown positive effects [25,26]. However, our interviewed GPs often felt burdened by the complaints and hardly talked about patient empowerment strategies. A ‘reluctance of clinical staff to provide active support for patient engagement’ is currently seen as the ‘biggest problem’ for patients with chronic disease [27]. It requires alteration of the professional role perception away from the ‘medical expert’ to the ‘facilitator’ [28] and training on how to apply these strategies in the consultation.

Second, GPs apply the idea of normal ageing as a yardstick for health and disease. Ageing is understood as a normal progressive process, a ‘non-disease’ [29]. A contrary concept states that biological ageing and pathological processes are similar, so that pathological ageing cannot be distinguished from normal ageing [30]. It is the underlying concept of ageing that has an impact on treatment decisions [31]: the theory of ‘normal ageing’ may paralyse treatment, whereas that of ‘complex damage’ may facilitate interventions and improvement.

Finally, GPs sometimes distance themselves from managing functional and social problems by arguing that such conditions are not part of their remit [3,14]. No doubt, the WONCA definition of general practice/family medicine covers these issues as part of person-centred, comprehensive and holistic care [32]. Our GPs have revealed an uncertain responsibility about this complex part of the role. On the one hand, expertise and time is needed to identify relevant functional and social problems [33]. On the other hand, complex intervention will involve delegation and co-ordination of care in a fully functional collaboration with other health professionals. A multidisciplinary approach to care with specialised staff, clear tasks and responsibilities is an area requiring health system changes and is recognised in Germany as well as in other countries [34,35]. Currently the lack of procedures and pathways that deal with these complex issues seems to impinge on the GPs’ motivation to act.

### Strengths and limitations of the study

In order to reproduce as realistic a situation as possible, GPs evaluated health problems of their own patients – and not hypothetical case vignettes. The structured nature of working through patient problems on lists, however, prevented a more abstract discourse on the perception of importance and priority setting. A further limitation is the small number of GPs interviewed and the omission of other health professionals. Since the study was conducted in Germany, health-system-related issues are not readily transferable to other settings.

## Conclusions

In the presence of multimorbidity a hierarchical management that considers priorities is often necessary. We found that GPs do prioritise health problems of their older patients. Medical risks and physical problems are judged to be important as are disabling and burdensome problems. Statements of active treatment, however, relate to medical risks and diseases, where effective monitoring and treatment strategies are readily applicable. Functional and social issues as well as psychological adjustment strategies for chronic progressive conditions receive too little attention. Future efforts should be directed to strengthening consultation strategies on coping with chronic disease. As the concept of ageing often hinders professional management, training should foster awareness of ageing and disability concepts. Interlocking co-operation systems with other health professionals are needed to ease the far-reaching complex workload of GPs inherent to the care of older patients.

## Abbreviations

Chi2-test: Pearson’s chi-squared test; Crit: Criterion; GP: General practitioner; KR-20 co-efficient: Kuder-Richardson Formula 20, measure of internal consistency for dichotomous variables; K-S-test: Kolmogorov-Smirnov-test, test for the normality of a distribution; SPSS: Statistical Package for Social Sciences; STEP assessment, Standard assessment for the Elderly in Primary Care, European Concerted Action, Project No SOC 95 200544 05 F03; TSH: Thyroid stimulating hormone.

## Competing interests

The authors declare that they have no competing interests.

## Authors’ contributions

UJW designed and oversaw the study, participated in the analysis and wrote the manuscript. JW and HDE conducted the interviews and analysed the text as did TS. BW conducted the tetrachoric factor analysis. EHP critically revised the manuscript and MLD oversaw the analysis procedure. All authors read and approved the final manuscript.

## Supplementary Material

Additional file 1 **The influence of “importance”,“nature of the problem” and personal characteristics on active treatment.** Bivariate analysis is used to demonstrate the relation of two variables, namely doctor-perceived importance and nature of a problem, with doctors’ statements of active treatment (first table). It is also shown to what extent patient and doctor characteristics relate to statements of active treatment (second table). Click here for file
