# Supplementary material for: What is important, what needs treating? How GPs perceive older patients’ multiple health problems: a mixed method research study
Source: BMC Res Notes. 2012 Aug 16;5:443. doi: 10.1186/1756-0500-5-443 (PMC3475051; doi:10.1186/1756-0500-5-443)
Supplement: Additional file 1 — The influence of “importance”,“nature of the problem” and personal characteristics on active treatment. Bivariate analysis is used to demonstrate the relation of two variables, namely doctor-perceived importance and nature of a problem, with doctors’ statements of active treatment (first table). It is also shown to what extent patient and doctor characteristics relate to statements of active treatment (second table). [file 1756-0500-5-443-S1.doc]

**The influence of “importance”,“nature of the problem”, and personal characteristics on active treatment.**

Bivariate analysis is used to demonstrate the relation of two variables, namely doctor-perceived importance and nature of a problem, with doctors’ statements of active treatment (first table). It is also shown to what extent patient and doctor characteristics relate to statements of active treatment (second table).

| **Problems with GP statements concerning treatability:** | all problems  N=199 100% | | no or vague treatment  N=124 100% | | | active treatment  N=75 100% | | *P *** |
| --- | --- | --- | --- | --- | --- | --- | --- | --- |
|  |  |  |  |  |  | |  |  |
|  |  |  |  |  |  | |  |  |
| important problems | 114 | 57% | 62 | 52% | 52 | | 72% | <0.01** |
| health domain |  |  |  |  |  | |  |  |
| physical problems | 90 | 45% | 37 | 30% | 53 | | 72% | <0.01** |
| pain | 12 | 6% | 10 | 8% | 2 | | 3% | 0.22 |
| senses | 12 | 6% | 12 | 10% | 0 | | 0% | <0.01 |
| functional disability | 18 | 9% | 17 | 14% | 1 | | 1% | <0.01** |
| housing & finances | 12 | 6% | 11 | 9% | 1 | | 1% | 0.03 |
| medication | 11 | 6% | 4 | 3% | 7 | | 10% | 0.10 |
| cognition | 5 | 3% | 3 | 2% | 2 | | 3% | 1.00 |
| mood | 18 | 9% | 13 | 11% | 5 | | 7% | 0.39** |
| lifestyle | 8 | 4% | 6 | 5% | 2 | | 3% | 0.72 |
| vaccination* | 13 | 7% | 11 | 9% | 2 | | 3% | 0.14 |
|  |  |  |  |  |  | |  |  |

# control, continue, change or start treatment

*vaccination is used as a reference unit for the multilevel logistic regression model

** chi2-test, otherwise Fisher’s exact test

| **Importance ratings**  **on the patient level^#^** | **Mean % of important problems** ± **st.dev. (t-test)** | | | | | | | |
| --- | --- | --- | --- | --- | --- | --- | --- | --- |
| 35 patients | for all patients | | for criterion 1* | | for criterion 2 | | *P* | |
| patient’s gender: 17 female ,18 male | 39% | ± 26 | 35% | ± 22 | 41% | ± 22 | 0.44 | |
| patient’s age: 19 patients < 80,  16 patients >= 80 years | 39% | ± 26 | 43% | ± 24 | 32% | ± 24 | | 0.17 |
| **Importance ratings**  **on the doctor level** | **Mean % of important problems** ± **st.dev. (t-test)** | | | | | | | |
| 9 doctors | all doctors | | for criterion 3** | | for criterion 4 | | *P* | |
| doctor’s gender: 4 female, 5 male | 41% | ± 30 | 41% | ± 15 | 38% | ± 28 | 0.84 | |
| doctor’s age: 5 doctors<50,  4 doctors >=50 | 41% | ± 30 | 43% | ± 27 | 35% | ± 14 | 0.67 | |
|  |  |  |  |  |  |  |  | |
|  |  |  |  |  |  |  |  | |

# K-S-test for Normal distribution: p= 0.2

* crit. 1: female patient or rather age <80 years, crit. 2: male patient or rather age>= 80.

** crit. 3: female doctor or rather age< 50 years, crit. 4: male doctor or rather age>= 50.
